# Research Ethics in the Age of Digital Platforms

**DOI:** 10.1007/s11948-023-00437-1

**Published:** 2023-04-25

**Authors:** José Luis Molina, Paola Tubaro, Antonio Casilli, Antonio Santos-Ortega

**Affiliations:** 1 GRAFO—Department of social and Cultural Anthropology, Universitat Autònoma de Barcelona, Bellaterra, Spain; 2 Laboratoire Interdisciplinaire Des Sciences du Numérique (LISN), Centre National de La Recherche Scientifique (CNRS), Université Paris-Saclay, Inria, France; 3 School of Telecommunications, Institut Polytechnique de Paris, Telecom Paris, France; 4 Department of Sociology and Social Anthropology, Universitat de València, Valencia, Spain

**Keywords:** Microwork, Crowdsourcing, Research ethics, Amazon mechanical Turk, Digital platforms

## Abstract

Scientific research is growingly increasingly reliant on "microwork" or "crowdsourcing" provided by digital platforms to collect new data. Digital platforms connect clients and workers, charging a fee for an algorithmically managed workflow based on Terms of Service agreements. Although these platforms offer a way to make a living or complement other sources of income, microworkers lack fundamental labor rights and basic safe working conditions, especially in the Global South. We ask how researchers and research institutions address the ethical issues involved in considering microworkers as "human participants." We argue that current scientific research fails to treat microworkers in the same way as in-person human participants, producing de facto a double morality: one applied to people with rights acknowledged by states and international bodies (e.g., the Helsinki Declaration), the other to guest workers of digital autocracies who have almost no rights at all. We illustrate our argument by drawing on 57 interviews conducted with microworkers in Spanish-speaking countries.

**Supplementary Information:**

The online version contains supplementary material available at 10.1007/s11948-023-00437-1.

## Introduction

The accelerated development of the digital economy is transforming the world and influencing ethical research standards. One apparent effect of this data-driven economy is the growing presence of digital platforms in everyday life (Srnicek, 2017). Digital platforms are businesses that connect different groups of user, notably clients and workers, and charge a fee for an algorithmically managed service based on Terms of Service agreements (ILO, 2022, p. 21). Among the two main types of digital labor platforms (those based on workers' locations and those based on remote digital work), in this paper we focus on the latter, specifically those offering online microtasks (or Human Intelligence Tasks, HITs) to so-called "microworkers." First popularized by Amazon with its "Mechanical Turk" (AMT) platform in the mid-2000s, microwork has grown over time and is now offered by many intermediaries, including international actors such as Appen, OneForma, Clickworker, and Microworkers, among others, a trend that will continue in the future (Cognilytica, 2020).

Other remote digital labor platforms, not considered here, hire specialized freelance workers for to perform complex tasks, possibly connecting them with potential clients (e.g., Upwork) that often have better working conditions. Microworkers usually perform three types of task: (a) training or verification tasks for artificial intelligence (AI) production (Tubaro et al., 2020), (b) content moderation or promotion, and (c) online studies. Indeed, scientific research increasingly relies on microworkers or "crowdsourcing" to conduct psychological and economic experiments, fill out surveys, and perform other data-generating tasks previously performed by college students or participants recruited by faculty labs and panel survey companies. All generalist platforms (like AMT) routinely offer survey options to their clients, and some, like Prolific, specialize precisely in this type of task.

The COVID-19 pandemic increased remote-working arrangements worldwide. This led unemployed workers and those with a preference for flexible work schedules to join the extant pool of microworkers, tightening competition (ILO, 2022). While earlier studies uncovered the reality of microwork in the United States, there is increasing evidence of its presence in countries like India (Gray & Suri, 2019), Indonesia (Lindquist, 2022), Brazil (Grohmann & Araújo, 2021) and Argentina (Miceli et al., 2020), as well as countries in sub-Saharan Africa (Anwar & Graham, 2022). Overall, the geography of digital platforms follows a North–South distribution, where a few companies from the Global North publish most of the microtasks to be completed by microworkers from the South (Couldry & Mejias, 2019). This geographical distribution explains why many microworkers typically work during the night or early morning, with a mean workload of 27 h per week, largely determined by the tasks available, and devoting at least 8 h of unpaid work to the computer screen for appropriate tasks. The mean earning estimate (including unpaid work time) ranges from $2.1 per hour (Berg et al., 2018) to $3.3 (ILO, 2022). Although these platforms offer a way to complement other sources of income or even make a living, notably for women with care duties, microworkers lack fundamental labor rights and basic safe working conditions (Posada, 2022; Wood et al., 2019).

At this point, our question is simple: what ethical issues arise when relying on microworkers to collect data for science, and how should researchers and research institutions address them? The literature usually recommends using microwork platforms for research including at most cursory references to ethics (Buhrmester et al., 2018). The few that pose ethical issues merely suggest ways to mildly mitigate them, such as informed consent, fair payment, detailed task description, swift task approval, and institutional identification.

In this paper, we argue that the ethical issues involved in scientific research relying on microwork platforms go beyond mere mitigation measures. A new conceptualization of "human participants" in a hybrid, digital world is necessary. Without this reconceptualization of research ethics, scientific practice fails to treat microworkers and in-person participants alike, producing de facto a double moral: one applied to people with rights acknowledged by states and international bodies (e.g., the Helsinki Declaration), the other to what we call "guest workers of digital autocracies" in order to point out the lack of regulation in the digital platforms’ labor sector. We illustrate this argument by drawing on 57 interviews conducted with microworkers in Spanish-speaking countries (TRIA project, see below).

The paper is organized as follows. First, we summarize the arguments provided by scholars in indexed journals about the use of microwork platforms for research. Second, we describe our research project and the methods used. Third, we describe microworkers' working conditions based on our interviews. Fourth, we discuss the ethical challenges microwork poses for current ethical research standards. We conclude by discussing the more general limitations of the current ethical procedures to address the emergent phenomena produced by the development of the digital economy.

## Low-Cost Science?

**Fig. 1:**
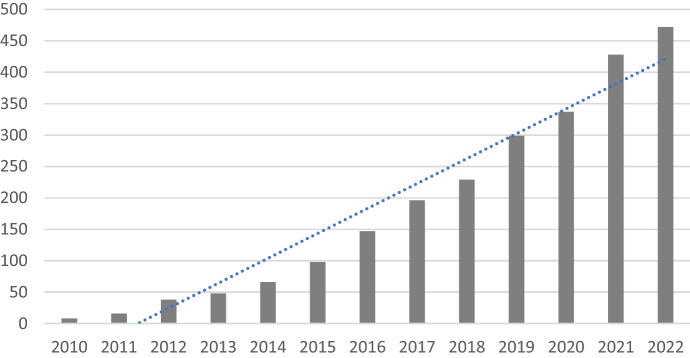
Number of articles published in Scopus from 2010 mentioning any of the digital platforms scored by Fairwork in the abstract (common names like “Rev”, “Prolific” or “Translated” were removed). Source: authors’ elaboration

Scientific research, especially behavioral experiments and machine-learning (Denton et al., 2021), relies increasingly on microwork to collect new data. AMT was the first microwork platform for researchers and is the most popular, in part because about three-quarters of "turkers" (AMT microworkers) are located in the United States of America, while the rest are in India and a very few in other countries (Paolacci & Chandler, 2014). Overall, the use of microwork in research has expanded over the last decade. Figure 1 shows the number of articles published in Scopus since 2010 (N = 2,509) that mention one of the digital platforms rated in Fairwork (https://fair.work) in the abstract.

The academic literature regarding "crowdsourcing" or microwork platforms falls into two broad categories: papers that recommend substituting traditional college students and laboratory panels with microworkers, and papers that acknowledge the existence of ethical issues in adopting this option. The former point out the advantages of low-cost, broad sampling possibilities, reliability, and effective project management, while the latter suggest mitigation measures or other ways to address the ethical issues involved in platform work.

### A "Revolutionary" Alternative to Conventional Methods of Data Collection

At the beginning of the ascendant curve of AMT use in research, Horton et al. (2011) presented the main arguments for abandoning expensive labs and college students as participants in favor of the "online laboratory": "Each of these replication studies was completed on MTurk in fewer than 48 h, with little effort required on our part”; "The cost was also far less than that of standard lab experiments, at an average cost of less than $1 per subject"; "We entirely avoided both the costs associated with hiring full-time assistants and the costs of maintaining a laboratory." Importantly, online experiments had the "same reliability" as more conventional ones.

These appealing arguments were successively confirmed by a long list of authors who praised AMT for providing "access to a massive subject pool available 365 days a year, freeing academic scientists from the boom-and-bust semester cycle" (Mason & Suri, 2012; Miller et al., 2017), selecting microworkers based on past approval rates at low cost but still high quality (Berinsky et al., 2012; Crump et al., 2013; Mortensen & Hughes, 2018; Strickland & Stoops, 2019). Authors also noted that even low payment was not an issue because microworkers *were not driven primarily by financial incentives* (Buhrmester et al., 2011; see also Paolacci & Chandler, 2014), nor by financial need, an argument elaborated recently by Moss et al. (2021): “our data show MTurk is a reasonably good opportunity for people to earn some extra money in their spare time and for behavioral researchers to collect some data.” However widespread, this view is at odds with the International Labor Organization’s report on digital platforms worldwide (ILO, 2022, p. 147), which estimates that leisure is the primary motivation of only 18% of microworkers, compared to 39% who aim to supplement other sources of income and 29% who seek job flexibility.

In the next section, we leverage our data to provide compelling evidence to support and extend the ILO’s views. Specifically, our findings reveal that, for two-thirds of our respondents, the primary reason for microworking is an urgent "need for money," which is consistent with the findings of Casilli et al. (2019) in France. Local conditions matter, and the focus of most of the previous literature on the United States may have masked situations of dire need that are much more widespread elsewhere. Indeed, 41% of our Latin American respondents agreed that "the political/economic situation of my country does not allow me to find a job" as their first, second, or third reason for microworking, which is significantly higher than the 22% reported by participants from Spain. To put this into perspective, the contrast between Spain and crisis-stricken Venezuela is even more striking, as 76% of respondents in Venezuela cited this reason.

Once the advantages of AMT were clear to researchers, attention shifted to biases not previously reported: for example, while microworkers performed *better* on online attention checks than conventional pool participants (Hauser & Schwarz, 2016), AMT microworkers exhibited *higher depression* rates than the general US population (Ophir et al., 2020). Interestingly, some authors have recently warned about an AMT "quality crisis" (Kennedy et al., 2020) because of alleged fraudulent respondents using Virtual Private Networks (VPN) to mask their IP connections, many of them being based in disadvantaged countries like India and Venezuela.

Although the literature about AMT is abundant compared with that on other platforms, and although it points out common traits of microwork-based research, it overlooks the most recent developments in the sector (Schmidt, 2022) and fails to account for the widening participation of the Global South, especially of non-English-speaking workers, notably in the Latin American case of Spanish-speakers examined in this article.

### Mitigation measures for ethical issues

The second stream of literature on microwork-based research has taken ethical issues into account from the outset. Behrend et al. (2011) reported that microworkers may be under pressure to complete surveys even if they want to opt out. They also noted that Informed Consent documentation was not always read carefully, or even made available, calling for more mindful approaches by researchers.

Another concern is "participant compensation." What would be a fair, ethical payment to microworkers? One option is to pay an amount as close as possible to the minimum wage in each country, and although some platforms, like Clickworker at the time of writing this article, make this recommendation, it is not a general rule (Pittman & Sheehan, 2016).

The right to *anonymity* that protects human participants is also mentioned as one of the ethical issues that are difficult to guarantee in microwork, even if workers’ profiles typically appear anonymized on platforms themselves (Lease et al., 2013):

[ATM] workers are not even protected by anonymity. If a worker ever left a product review or even assigned a "star" rating to a product anywhere in the Amazon universe, their user information could be linked to their MTurk account. This is particularly problematic if a Turkworker uses his or her real name to rate products on Amazon because researchers could learn an individual's gender and possibly other information. Indicating that responses may not be anonymous on the consent agreement allows workers to choose whether they wish to continue with the HIT or not. (Sheehan, 2018)

Researchers suggest that these ethical issues can be addressed with mitigation measures like transparency about employers’ identities, better task description, swift communication with microworkers, avoiding blocking privileges, privacy protection, and fair payment (Gleibs & Albayrak-Aydemir, 2022; Goodman & Paolacci, 2017).

## Data and Methods

In what follows, we draw on the interviews conducted online within the framework of the project *The Labor of Artificial Intelligence: Ethics and Governance of Automation (TRIA)*^1^. The broader goal of the project was to document the living and working conditions of platform microworkers in Spain and Latin America. We expected this under-researched geographical and linguistic setting to reveal aspects that previous studies, mostly focused on the English-speaking world, might have missed, and we also aimed to compare higher- with middle-to-lower income countries. Latin America also includes the exceptional case of Venezuela, where an extremely severe economic and political crisis has pushed many people toward international platform work (Posada, 2022).

Firstly, we posted a web survey between mid-December 2020 and early February 2021, achieving 1,134 complete responses from Latin America and 343 from Spain (N = 1,477, see Supplementary Material). The survey was entitled "Microtrabajo en países Hispanohablantes—Microwork in Spanish-speaking countries", was in Spanish and lasted 29 min on average. It included over a hundred questions covering basic socio-demographic information, education and skills, family situation, professional status and experience, income, internet usage, practices of micro-tasking on platforms, and social relationships. To recruit participants, we played the same game as those researchers who use microwork to collect data: we became clients, or in platform terminology "employers," on one of them, Microworkers.com. Fielding surveys as remunerated tasks on platforms is practically the only way to constitute a sizable and diverse sample of microworkers, as they are geographically dispersed and largely invisible to outsiders due to their working from home as one-person micro-enterprises. The reason for selecting Microworkers.com was its wide international base, more diverse than AMT (Berg et al., 2018). By acting as platform employers, we could also gain first-hand experience of what concrete, ethically relevant issues arise when hiring microworkers. For example, we aimed to be maximally inclusive and did not impose any prerequisites, except accepting the consent form and the policy of personal data protection (GDPR compliance, see www.gdpreu.org). We consulted the literature, the platform’s managers and experienced colleagues to establish a remuneration slightly above the minimum wage but without creating distorted incentives, also taking into account differences in the cost of living and volatile exchange rates across the eighteen countries in our sample. We settled for a payment of $5.50 to participants in Latin America and of $7 in Spain.

Secondly, between March and September 2021, we conducted follow-up online interviews with a sub-sample of survey participants, also in Spanish, to better appreciate the meanings that they gave to their responses and their lived experience. Their average duration was about 45 min. Interviewees from Latin America were rewarded with $7 and those from Spain with $8.50. Most interviewees held a degree and over one fourth were college students, some in engineering and computer science, who had combined online courses with microwork during the COVID-19 pandemic. High levels of education are a globally shared trait of microworkers (Berg et al., 2018).

Platform micro-tasks constitute the main source of income of four out of five Venezuelan respondents in our sample, of two out of five non-Venezuelan Latin Americans, and of only one out of five Spaniards. This is an important difference with respect to some previous studies on AMT: in the Global North, microwork earnings usually complement other sources of income, but this activity is often the most important source of income in the South.

While not constituting the majority of our web-survey respondents, Venezuelans and other full-time microworkers were particularly keen to participate in the interviews and are over-represented in our qualitative sample (Table 1).

**Table 1:** Descriptive statistics of interview participants, in absolute numbers. N = 57. Interpretation: 21 interviewees reside in Venezuela. Source: authors' elaboration

Country of residence		Country of birth	
Venezuela	21	Venezuela	25
Colombia	10	Colombia	9
Peru	5	Peru	5
Argentina	5	Argentina	4
Ecuador	5	Spain	4
Spain	5	Other	10
Other	6		
Age		Education	
18–24	19	Secondary school	4
25–34	20	Post-secondary or short HE	14
35–44	14	Bachelor	33
45–54	3	Master's or PhD	6
55 or more	1		
Marital status		Household size (incl. respondent)	
Single	28	1 person	3
Married	12	2 people	8
Cohabiting couple	14	3–4 people	24
Divorced/legal separation	2	5–6 people	14
Other/don't wish to answer	1	7 or more people	8
Have children		Gender	
Yes	23	M	43
No	34	F	14
Professional status		Platform earnings in last 3 months (in US$)	
Employee with permanent contract	9	0	1
Employee with temporary contract	8	1–50	9
Independent worker	12	51–100	12
Other working situation	3	101–200	10
Student	18	201–500	18
Retiree	1	501 or more	7
Unemployed	6		
Platform income as main income			
Yes	35		

While our analysis relies primarily on interviews, we cross-checked our findings with the results of the questionnaire to provide a more comprehensive picture of microworkers’ experiences and working conditions.

## Microworkers are Human Beings After all

### Motivations and Working Conditions

In situations of hyperinflation such as Venezuela’s, where the monthly minimum wage fell to as little as $3.5 at the time of our interviews, platform microwork is attractive because it pays in hard currency through systems such as Paypal and Airtm (with a fee), or even in cryptocurrencies, and because, when working full-time, microworkers can earn as much as $150-$200 per month. Also, working at home with some flexibility is evaluated positively, especially by women with domestic responsibilties. Nonetheless, research also shows that microwork reproduces and exacerbates gender inequalities. The low proportion of women in our sample (about one third overall, higher in Spain than in Latin America), comparable to the findings of Berg et al. (2018), mirrors their participation in conventional labor markets. Especially in countries in which microwork is often the main source of income for a household, like Venezuela, it is mainly practiced by men, while women perform tasks of lower complexity, and their careers are relegated to a secondary role. For the women who do microwork, Tubaro et al. (2022) show that “inequalities in both the professional and domestic spheres turn micro-tasking into a ‘third shift’ that adds to already heavy schedules”. The case of an Argentinean single mother (25–34 years old) with two children 10 and 6 years old illustrates this situation: she devotes 3 h to microwork when they go to school. Then, when they return around 11:00, she keeps working with frequent interruptions until nightfall, when more tasks come in. At home, she is always working, including at the weekend. "It is not easy to organize, but you do what you can."

Typically, the microworkers interviewed for this research work either in their bedroom, combining a small desk and a laptop (80% of questionnaire respondents) with the cell phone (84%), or at the dining room table. In Venezuela and some other parts of Latin America, internet connection often fails during peak hours or does not allow tasks to be performed that consume a lot of bandwidth. Workers usually have several tabs opened in their browser, search for tasks on multiple platforms, and refresh the screen frequently but not continuously to avoid being mis-labeled as bots and blocked by the system. They prefer comparatively "well-paid" tasks that can be completed quickly (2–10 min) to avoid submission rejections.

Tasks payments are measured in *cents* more than in dollars, and it is hard to make a living out of them:

(…) for example, that of identifying objects. Between 30 and 50 cents (for 5 min of work). (*Male, Mexican, 35-44 years old, living with his partner*).

It is very little; they pay you pennies. Put those cents here in Argentina. It's not money either, but well, earning pennies here is not the same as earning pennies, I imagine in Europe (…) but what they pay is very little, there are tasks for 10 cents, 15 cents." (*Male, Argentinean, 35-44 years old, single*).

18, 19 hours [of work] for about 8 to 10 dollars. It is tiny. The truth for the economy is not even the basic salary of my country, the truth. (*Male, Peruvian, 25-34 years old, married*).

Competition is high, especially for Venezuelan microworkers, who are particularly numerous (over 100,000 on one platform, according to its managers, at the time of conducting the study) and who, at the time we conducted the interviews, were facing a quota that limited their participation to 10% of each task submission on a platform. These working conditions and the time difference between employers and microworkers result in working at night or in the early morning:

So if the work is for a hundred people, only 10% of the people who are in Venezuela can do it. So you have to do the tasks very quickly. Thus, from six to eight in the morning many tasks come out, later they are sporadic (...). And wait … So in the group we warned each other. Look, a task came out! So you're already on the phone, ah, the task came out, you run to the computer if you are far away and you want to finish it and you run out and go and do it. (…) And then at midnight, the tasks that you could not do in the morning are released (*Male, Venezuelan, 35-44 years old, single*).

I get up at six, but there are people who get up at four in the morning. Others who get up at three. And they start until twelve, at twelve they sleep for two hours and (...) they continue their working day at two (*Male, Spanish living in Equador, 25-34 years old, living with his partner*).

### Personal Data Work

On most platforms, microworkers are anonymous to clients who only see their registration number, country of residence, success rate, and sometimes specific skills such as knowledge of some given language. Platforms do not usually allow workers and clients to share emails or other contact information. However, as discussed above, in many cases (almost 30% in our sample) microworkers need to use their personal, sometimes uniquely identifiable data to perform the tasks. For instance, development of AI emotion recognition algorithms may need fresh human faces:

The employer was German. He asked you to record yourself with your cell phone and start replicating the images that he showed you, for example, and it was: [face of] surprise, fear, crying. […] Depending on how your reaction was, how you managed it, he accepted you or not" (*Male, Argentinean, 18-24 years old, married*).

Content promotion typically requests "likes," positive reviews of products or services, and recommendations through social media sites, which require registration through one’s own account:

Those tasks pay a lot. Sometimes I have been paid up to a dollar. For writing a review of a company product. I mess with my profile or with the profile of another person and this... I analyze a product, I see the good, the bad, the better or worse. And I write them a review about the product (*Male, Venezuelan, 25-34 years old, single*).

I use my real account because they ask that it be a real account with real followers, so I have to use my own, which is the one I have been managing for a long time and well. They ask for a certain number of followers for a certain amount of time in the account, for example, sometimes they ask you to have more than 50 followers on Instagram, sometimes they ask you to have 200, sometimes 500 (*Female, Venezuelan living in Argentina, 18-24 years old, single*).

Finally, microworkers complete surveys and experiments the best they can because these are usually better paid than other tasks or maintain a good ranking score and make them eligible for the future. Again, they may have to share some personal data depending on the contents of the survey, although there are often safeguards such as informed consent forms. In fact, researchers cannot delete microworkers’ logs or personal data provided for the specific survey or experiment when they are stored in the platform’s databases. Overall, it is difficult for microworkers to keep track of the processing of their data or its potential consequences.

### Platform Power Asymmetry

Platforms control microworkers almost totally, while transferring power to clients. First, microworkers must accept the Terms of Service unconditionally and usually have to provide some formal proof of identity to be paid. An excerpt from Amazon Turk’s "Agreement" illustrates this point:

We may terminate this Agreement, terminate or suspend your account and access to the Site, or remove any Task listings immediately *without notice for any reason* [italics added]. Upon any termination or suspension of this Agreement, your right to use the Site will cease, and you will not be able to retrieve any information related to your account. [https://www.mturk.com/participation-agreement]

Second, the quality and quantity of tasks offered to microworkers depend upon the rate of their successful task submissions in the past, which constitute a "reputation" indicator attached to their profile. For instance, employers may submit a task only available to microworkers with a 95% success rate or higher, a recommendation sometimes made to achieve quality in data collection (Peer et al., 2014). However, this practice may entail validity problems by reducing diversity (Robinson et al., 2019). A common complaint is that tasks are sometimes rejected (and left unpaid) without further explanation, a trait that Gray and Suri dubbed “algorithmic cruelty” (2019), whereby the transaction costs of human-platform interactions fall disproportionately on the workers. In some cases, the algorithmic supervision and the internal competition enacted in the ranking lead microworkers to justify platform discipline.

[my rating is high] because of the time I've had on the platform, so now I know how to do most tasks. But there are many people who are just starting out, who simply make a lot of mistakes (...) They are very bad, it does not help them, or they take a screenshot of something else that the employer is not asking them to do. So, experience is what gives you a good, good rate and thank God that when I started [a friend] helped me a lot so I wouldn't make a mistake. (*Male, Spaniard living in Ecuador, 25–34 years old, living with his partner*).

Third, there are no payment floors. Literally, the employer can pay anything, refuse to pay any worker, and even block further participation.

Segmentation of projects into tasks, client anonymity, international scope, and a lack of background information can be described as a process of alienating microworkers from their work, ignoring the specific value they produce for others. The classic work of Seeman (Seeman, 1959) identifies five alternative meanings of alienation: powerlessness, meaninglessness, isolation, normlessness, and self-estrangement. At least the first three meanings apply to our case, suggesting that the classic conceptualizations of earlier forms of capitalism and the proletariat are still useful for understanding this phenomenon.

## Crowdsourcing Research Versus the Helsinki Declaration and the Belmont Report

Biomedical research standards set up after World War II influenced the ethical review practices currently followed by Institutional Review Boards (IRBs) or Research Ethics Committees (RECs) (Molina & Borgatti, 2021). For instance, the concept of prior, understandable, and free consent was formulated in the Nuremberg Code in 1947 (Kottow, 2007), and later expanded in the Declaration of Helsinki (1964–2000) and the Belmont Report (1979), which proclaimed the participant's right *to withdraw at any time without reprisal* (Art. 26 and Section C.1 respectively).

Following these ethical standards (see Israel, 2015), we argue not just that microworkers cannot be considered regular "participants," but that recruiting microworkers entails serious ethical issues that researchers should consider adequately. Table 2 compares the list of rights to be preserved (left column) with the contrasts in the microworkers' situations (right column):

**Table 2:** Microwork from the point of view of IRBs/RECs. Source: authors’ elaboration

IRB/REC: ethics requirements	Microwork: crowdsourcing
Freedom to "participate"…	“Amateurs” are a minority. Microworkers need to do Human Intelligence Tasks (HITs) to make ends meet or to complement other sources of income
… fair "compensation" (not payment)	Low "standard" wages for tasks
… opt out without consequences	Withdrawal, especially for less experienced workers, implies losing an opportunity to improve their success score (and be eligible for future better-paid tasks)

Taking the current ethical review standards seriously indicates that IRBs and RECs cannot consider microworkers as “free participants” or “one-time participants” who are compensated and not paid, with the right to opt out without further explanation or consequences. Yet these requirements still apply to regular, in-flesh participants.

The codes mentioned above pay special attention to research conducted with vulnerable groups. The Belmont report says (emphasis added):

One special instance of injustice results from the involvement of vulnerable subjects. Certain groups, such as racial minorities, the *economically disadvantaged*, the very sick, and the institutionalized may continually be sought as research subjects, owing to their *ready availability* in settings where research is conducted. Given their *dependent status and their frequently compromised capacity for free consent*, they should be protected against the danger of being involved in research solely for administrative convenience, or because they are easy to manipulate as a result of their illness or *socioeconomic condition*. (Section C.3)

In sum, current ethical review standards pose serious doubts on research with microworkers, even after incorporating mitigation measures. According to the Belmont Report, availability and low cost do not justify recruiting microworkers as "human participants" in ethical scientific research. Despite this fact, IRBs and RECs regularly approve this type of research because the outsourcing process involved in “crowdsourcing” is also a way to avoid *responsibilities*, one of the main concerns of any bureaucratic organization, including IRBs and RECs (see, for instance, van Den Hoonaard, 2011, p. 45). In addition, if the platform has a lawful personal data-protection policy, the perception of the vulnerability of microworkers by IRBs and RECs may be masked by their formal “freedom” to accept the Terms of Service (including personal data policies) and to “choose” tasks.

We must therefore admit that the current ethical frameworks are not suited for this reality and that it is necessary to conceptualize platform microwork in ways that adequately assess its moral dimension, especially when microworkers from lower-income countries contribute to research projects.

## Citizens Versus Guest Workers in “Digital Autocracies”

The research ethics standards developed from World War II onwards apply to in-flesh citizens whose rights are acknowledged and enforced by national states and international institutions, but not to microworkers who operate in a globalized, online-only labor market. We argue that microworkers should be conceptualized as “guest workers” (Aytes, 2012) in “digital autocracies” which not only shape economic interactions but extend their normative functions to affect members’ fundamental rights. The largely unregulated sector of digital work allows platforms to impose their own rules, for instance, limiting association or the right to be tried by a jury. The Terms of Service of AMT, a major actor in this sector, illustrate this point:

We each agree that any dispute resolution proceedings will be conducted only on an individual basis and not in a class, consolidated, or representative action. If for any reason a claim proceeds in court rather than in arbitration, *we each waive any right to a jury trial* [emphasis added]. [https://www.mturk.com/participation-agreement].

In this vein, Floridi (2020) stresses the political dimension of these private corporations by quoting the opening statement of Chairman David N. Cicilline on July 29, 2020 at the hearing of the House Judiciary Subcommittee on Antitrust, Commercial and Administrative Law about “Online Platforms and Market Power”:

[…] Because *concentrated economic power also leads to concentrated political power*, this investigation also goes to the heart of whether we, as a people, govern ourselves, or whether we let ourselves be governed by private monopolies. American democracy has always been at war against monopoly power. […] Their ability to dictate terms, call the shots, upend entire sectors, and inspire fear represent the powers of a private government. Our founders would not bow before a king. Nor should we bow before the emperors of the online economy” [emphasis added].

Zuboff (2019, location 15,930) compares the capacity of new digital businesses to impose their rules in the uncharted territory created by technology development with the sovereignty claim of the Castilian King over America:

Like the *adelantados* [first Spanish conquerors of America] and their silent incantations of the *Requirimiento* [Declaration of Castilian King’s sovereignty upon the new territory], surveillance capitalism operates in the declarative form and imposes the social relations of a premodern absolutist authority. It is a form of tyranny that feeds on people but is not of the people. (…) It replaces legitimate contract, the rule of law, politics, and social trust with a new form of sovereignty and its privately administered regime of reinforcements.

Following Zuboff's argument, the unilateral capacity of digital platforms to regulate this new space of value creation through a combination of organization, technology, and human labor allows them to impose conditions on their new workforce in the key domains of a) identity, b) working conditions, and c) means of payment, prerogatives usually reserved to governments. The AMT microworkers’ forum "Turker Nation" illustrates this point vividly.

These “digital autocracies” extract value from microworkers through their ability to mediate between cheap labor and computers, a process known as "heteromation" (Ekbia & Nardi, 2017), as well as through the appropriation of microworkers' rights to privacy and personal data protection. Microworkers not only use their "human" neurobiological capacities but also their political existence as nominal citizens and their social connections to provide value for platforms. Despite progress in many countries to regulate on-demand labor platforms (delivery, transportation, and personal services) and to improve working conditions, the ubiquitous nature of online-only labor platforms makes regulation challenging to achieve (ILO, 2022). Nowadays, microwork platforms primarily draw on self-regulation recommendations (Martin et al., 2017) like those promoted by FairWork (https://fair.work).

The hybrid political dimension of microwork sheds light on the ethical dimensions of resorting to digital platforms as sources of research data. In this vein, microworkers should be considered *prima facie* vulnerable populations, and flagged as such by ethics review committees. This conceptualization of microworkers has consequences for ethics criteria:

1. Researchers should *avoid* relying on microwork as the default option, and should do so only when the specific needs of their study require it. Uncritical reliance on microworking platforms leads to public and private research funding legitimating, and enforcing the existence of, unregulated digital autocracies.
2. As in the case of animal experimentation, researchers should justify any need to use microwork for collecting data and demonstrate that there are no suitable *alternatives*. Note that cost arguments are not acceptable when discussing the inclusion of vulnerable populations.
3. The mitigation measures proposed by the literature (informed consent, fair payment, good task description and research project background, swift task approval, and open and direct communication) should grant that the microworker’s success rate and personal data protection rights are fully respected.
4. International assessments of digital platforms’ fair work arrangements (https://fair.work/en/ratings/cloudwork/) should be considered when selecting the digital platform.

In addition, IRBs/RECs must acknowledge that millions of people engage routinely with digital platforms or panel companies driven by economic motivations instead of voluntarily collaborating with research projects. Accepting that “professional” participants are part of research projects and that the concept of “compensation” is no longer generally applicable to an extensive range of circumstances is an exercise in realism. That said, the issue remains that of what a "fair payment” is. We do not propose one-size-fits-all recommendations because our experience suggests that workers with better organization and technical equipment often monopolize tasks that pay relatively well (or even that just pay their country’s minimum wage, see Verma et al., 2021), crowding out weaker workers. Conversely, payments cannot be too low. Workers use platforms to earn money and are sometimes in dire financial need: to contribute to research, they must forgo some other job. Compensating them thus means that researchers should play the role of full-fledged—albeit temporary—employers and pay at least the minimum wage in line with labor regulations. At this point, it is essential to rely on researchers’ ethical commitment to sound science to find the right balance between fair pay and the need to avoid bias in each case.

## Conclusion and Limitations

In this work, we conceptualize microworkers as guest workers of unregulated, digital autocracies. Although not all microworkers are equally vulnerable, and many of them derive economic and personal advantages from this activity, microworking platforms extract value by denying basic labor and human rights to their guest workers. Ethically sound scientific research cannot apply a double moral standard for in-person participants and hybrid microworkers, as happens nowadays. We suggest that researchers and ethics review committees should consider microworkers primarily as vulnerable populations, should avoid including them by default, and should set all the necessary measures to guarantee their fundamental rights. This stance is coherent with the Belmont report’s principle of “respect for persons,” which states that “The principle of respect for persons thus divides into two separate moral requirements: the requirement to acknowledge autonomy and the requirement to protect those with diminished autonomy.”

Microworkers’ willingness to participate in often better-paid academic tasks does not justify their recruitment. Likewise, the fact that many of them agree to use their personal data as an asset providing value to the platform does not justify the promotion of this business model.

We must acknowledge that the negotiating capacity of researchers and IRB/REC with these often large multinational companies is almost null. Many of them hire researchers who routinely publish articles in academic journals using their rich, private databases of microworkers from which to draw samples and highlighting the advantages of this resource to boost scientific projects and publications. Their visibility also raises concerns about the limits of academic research and academic journals to offer independent and peer-reviewed knowledge to society (Michaels, 2020; Michaels & Monforton, 2005). All in all, the phenomenon of microwork poses deep concerns about the growing societal inequalities that arise in today’s digitized and globalized world, as well as highlighting the need to update our ethical framework for scientific research.

## Supplementary Information

Below is the link to the electronic supplementary material.

Supplementary file1 (DOCX 20 KB)

## Footnotes

1 The project TRIA was funded by the CNRS, MSH Paris Saclay. IP: Paola Tubaro.
