# Supplementary material for: Research Ethics in the Age of Digital Platforms
Source: Sci Eng Ethics. 2023 Apr 25;29(3):17. doi: 10.1007/s11948-023-00437-1 (PMC10127972; doi:10.1007/s11948-023-00437-1)
Supplement: Supplementary file 1 — Supplementary file1 (DOCX 20 KB) [file 11948_2023_437_MOESM1_ESM.docx]

**Table**: Descriptive statistics about the surveyed population, in percentage. N = 1477. Interpretation: 23% of respondents are based in Spain.

| **Country of residence** | | **Country of birth** | |
| --- | --- | --- | --- |
| Spain | 23 | Spain | 17.3 |
| Colombia | 17.8 | Colombia | 16.5 |
| Venezuela | 10.7 | Venezuela | 15.6 |
| Mexico | 10.7 | Mexico | 10.6 |
| Argentina | 10.4 | Argentina | 9.8 |
| Peru | 8.4 | Peru | 7.9 |
| Ecuador | 5.2 | Ecuador | 4.7 |
| Dominican Republic | 2.9 | Dominican Republic | 3 |
| Bolivia | 2.8 | Bolivia | 2.7 |
| Other | 8.2 | Other | 11.9 |
|  |  |  |  |
| **Age** | | **Education** | |
| 18-24 | 41.9 | Primary school or less | 1.5 |
| 25-34 | 34.4 | Secondary school | 11.1 |
| 35-44 | 16.6 | Post-secondary or short HE | 24.1 |
| 45-54 | 5.6 | Bachelor | 51.7 |
| 55 or more | 1.5 | Master's or PhD | 8.9 |
|  |  | Don't know / don't wish to answer | 2.7 |
|  |  |  |  |
| **Marital status** | | **Household size** | |
| Single | 60.1 | 1 person | 5.4 |
| Married | 18.8 | 2 people | 12.2 |
| Cohabiting couple | 15.8 | 3 - 4 people | 46 |
| Divorced / legal separation | 2.5 | 5 - 6 people | 28.6 |
| Widow | 0.2 | 7 or more people | 7.7 |
| Other / don't wish to answer | 2.7 |  |  |
|  |  |  |  |
| **Have children** | | **Gender** | |
| Yes | 28.3 | M | 66.9 |
|  |  | F | 32.7 |
|  |  | Other | 0.4 |
|  |  |  |  |
| **Professional status** | | **Platform earnings in last 3 months (in US$)** | |
| Employee with permanent contract | 16.8 | 0 | 32.6 |
| Employee with temporary contract | 7.6 | 1-50 | 29.1 |
| Independent worker | 11.1 | 51-100 | 13 |
| Other working situation | 9.5 | 101-200 | 9.9 |
| Student | 33.6 | 201-500 | 9.5 |
| Retiree | 0.6 | 501 or more | 5.8 |
| Unemployed | 14 |  |  |
| Inactive | 6.8 |  |  |
|  |  |  |  |
| **Platform income as main income** | |  |  |
| Yes | 38.1 |  |  |
